# Elevated level of D-dimer increases the risk of stroke

**DOI:** 10.18632/oncotarget.23367

**Published:** 2017-12-18

**Authors:** Jing Zhang, Yanlin Song, Baoyin Shan, Min He, Qingqing Ren, Yunhui Zeng, Zhiyong Liu, Hao Liu, Jianguo Xu

**Affiliations:** ^1^ Department of Neurosurgery, West China Hospital, Sichuan University, Chengdu 610041, PR China; ^2^ West China School of Medicine, West China Hospital, Sichuan University, Chengdu 610041, PR China

**Keywords:** D-dimer, stroke, risk factor

## Abstract

The aim of this study was to systematically evaluate the association between D-dimer level and the risk of stroke through performing a meta-analysis. PubMed, Web of Science, EMBASE and Cochrane Library were searched for potentially eligible literature. Prospective observational studies or case-control studies were included. The study characteristics and relevant data were extracted. Hazard ratios (HRs) or odds ratios (ORs) with 95% confidence intervals (CIs) were pooled to estimate the association between D-dimer level and the risk of stroke. Seven prospective studies with 22,207 patients and three case-control studies with 2,248 patients were included. For the prospective studies, the pooled HRs of higher D-dimer level for all types of stroke, ischemic stroke and hemorrhagic stroke were 1.55 (95% CI, 1.28- 1.87), 1.62 (95% CI, 1.18-2.22) and 1.30 (95% CI, 0.63-2.68), respectively. The pooled HRs per SD increase in log D-dimer for all types of stroke, ischemic stroke and hemorrhagic stroke were 1.16 (95% CI, 1.06-1.26), 1.11 (95% CI, 1.03-1.21) and 1.11 (95% CI, 0.95-1.30), respectively. For the case-control studies, the pooled OR of higher D-dimer level for acute ischemic stroke was 2.06 (95% CI, 1.08-3.96). No significant publication bias was found in the meta-analysis. In conclusion, our results suggested that higher D-dimer level was associated with higher risk of stroke, especially ischemic stroke.

## INTRODUCTION

Stroke is one of the leading causes of mortality and disability worldwide [1]. In China, stroke is the leading cause of mortality and adult disability, with the annual stroke mortality rate of approximately 157 per 100,000 people [2]. Besides, stroke has a considerable impact on healthcare expenditures [2]. Ischemic stroke, characterized by the disruption of cerebral blood flow, accounts for approximately 80%-85% of all strokes [3]. Timely thrombolytic therapy is an effective treatment for acute ischemic stroke, however, the therapeutic window is very narrow [4]. Intracerebral hemorrhage (ICH) represents approximately 10% to 20% of all strokes [5]. ICH is characterized by high rates of mortality and disability, and currently little effective therapeutic strategies are available [6]. Primary prevention of stroke is essential and mainly includes the control of risk factors and preventive antithrombotic treatments [7]. Apart from the well-documented and modifiable risk factors, such as, physical inactivity, hypertension, dyslipidemia, diabetes mellitus and cigarette smoking, it is worthwhile to detect new potentially modifiable risk factors and control them at an early stage [7–9].

D-dimer, a circulating peptide degradation product of cross-linked fibrin, is formed during activation of the coagulation system [10]. Higher levels of D-dimer indicate more systemic fibrin formation and a tendency for increased thrombosis [11]. It has been proposed as a marker of the state of coagulation and fibrinolytic systems [12]. There are numerous assays for D-dimer measurement [13]. Enzyme-linked immunosorbent assay (ELISA) could provide highly sensitive quantitative results and has been used as the reference method [14]. However, it is a time-consuming procedure [13]. Immunoturbidimetric assay is another measurement of plasma D-dimer, and has shown comparable sensitivity and specificity [13, 15]. Immunoturbidometric assay has also shown acceptable interassay coefficient of variation [16–18].

In clinical use, measurement of D-dimer has become an essential element to exclude deep vein thrombosis and pulmonary embolism [19, 20]. It is also used in the diagnosis and monitoring of coagulation activation in disseminated intravascular coagulation [11]. D-dimer is a predictive marker for cardiovascular disease. It has been suggested that elevated D-dimer level not only is a risk factor of coronary heart disease [21, 22], but also predicts worse outcome in cardiovascular diseases [23, 24]. In patients with stroke, increased D-dimer level was found to be associated with worse functional outcome [9, 25, 26]. Many researchers have also investigated the relationship between D-dimer level and the risk of stroke. Some concluded that higher D-dimer was associated with higher risk of stroke [10, 27, 28], but some investigators did not reach such conclusions [29, 30]. For example, in a case-control study by Kaplan et al. [29], the authors found that D-dimer was not an independent predictor of ischemic stroke. In a prospective study, in which the subjects were followed for a mean of 17 years, the researchers concluded that the predictive ability of D-dimer was modest and clinical utility remained uncertain [30]. Due to the controversy, a meta-analysis was designed to systematically review the association between D-dimer level and the risk of stroke, and to pool the evidence together to reach a conclusion.

## RESULTS

### Literature research

**Figure 1:**
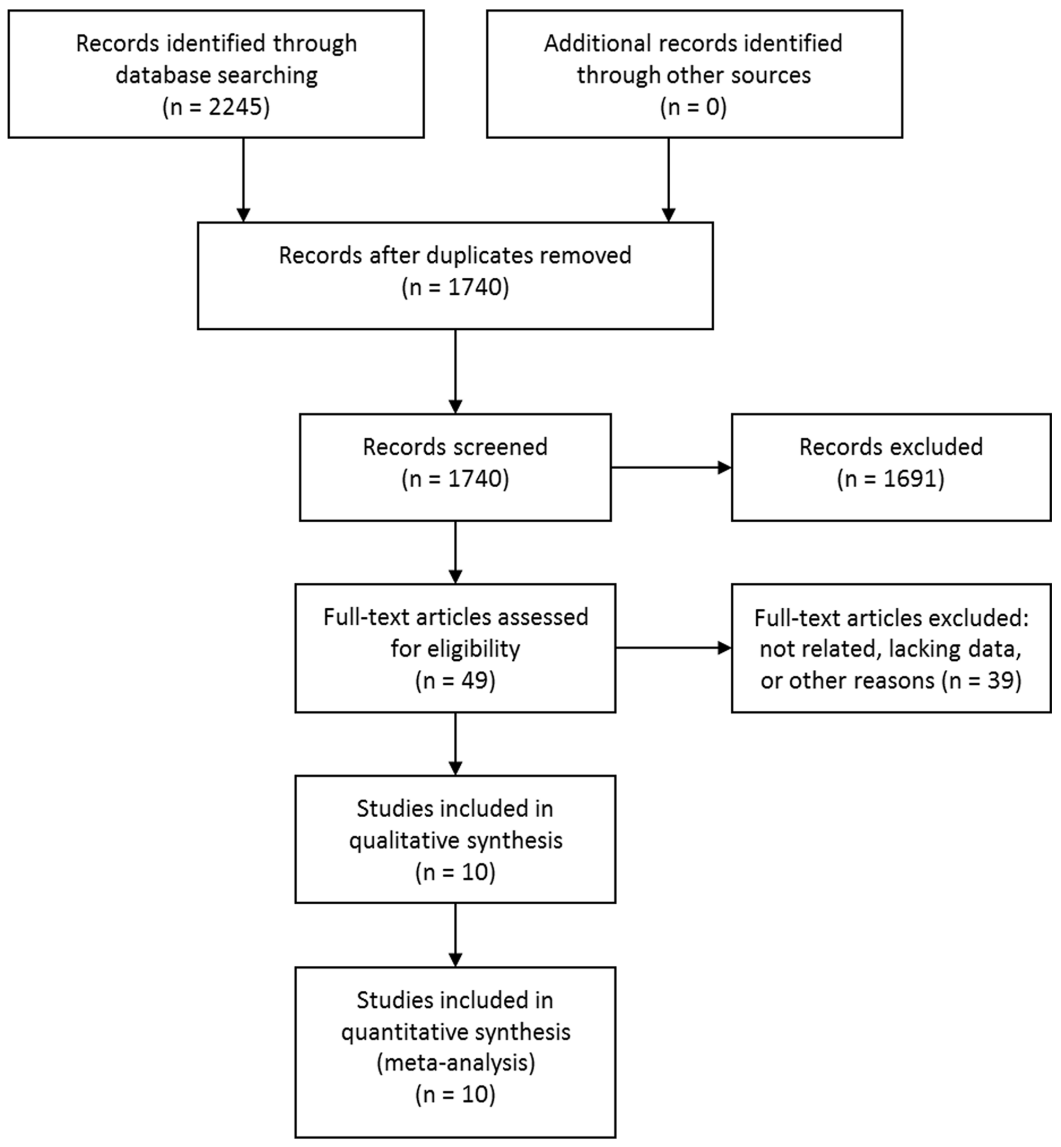
Selection process of studies

The initial database searching identified 2245 studies. No additional studies were identified through other sources. After removing duplicates, 1740 studies were screened by titles and abstracts. According to the predefined inclusion and exclusion criteria, 1691 studies were excluded. The rest 49 studies were evaluated in full text and 37 were further excluded due to unrelated, lacking enough data or other reasons. Two articles were from the same study cohort [28, 30], and the study with shorter follow-up time was excluded [28]. Another two articles were also from the same study cohort [29, 31], and the study with smaller sample size was excluded [31]. Eventually, 10 articles [10, 16–18, 29, 30, 32–35] met the inclusion criteria and were included. Of note, although two studies were from the same study cohort [10, 16], one study examined the risk of all types of stroke and the other examined the risk of intracerebral hemorrhage, so they were not pooled together during the analysis and were both included. The study selection process was shown in Figure 1.

### Study characteristics

The basic characteristics of the 10 included studies were shown in Table 1. Among them, seven were from prospective studies. The seven prospective studies were published from 2005 to 2017, with 3 from UK, 3 from USA and 1 from Italy. A total of 22,207 patients were included (mean 3,172, median 1,750). The seven studies examined the HR of higher D-dimer level for all types of stroke, ischemic stroke or hemorrhagic stroke. All the HRs were calculated form multivariate analyses.

**Table 1:** Characteristics of the included studies

Author	Year	Country	N (F/M)	Mean age	Study	Disease	Follow-up time	Measure methods	Cut-off value	Estimate	Adjusted HR/OR
**Prospective studies**											
Tzoulaki	2007	UK	1592 (783/809)	64.9	The Edinburgh Artery Study	Stroke	mean 17 yrs	ELISA	tertile	HR	Yes
Wannamethee	2012	UK	3358 (0/3358)	68.4	The British Regional Heart Study	Stroke	mean 9 yrs	ELISA	tertile/per SD	HR	Yes
Zakai	2017	USA	1750 (—/—)	—	The REGARDS Study	Stroke	median 5.8 yrs	ITA	quintile	HR	Yes
Di Castelnuovo	2014	Italy	832 (550/282)	—	The EPICOR Study	Stroke/HS/IS	mean 9 yrs	ITA	quartile/per SD	HR	Yes
Folsom	2016	USA	11415 (6632/4783)	59.8	The ARIC Study	Stroke/HS/IS	median 18 yrs	ITA	quintile/per SD	HR	Yes
Smith	2005	UK	2208 (0/2208)	56.9	The Caerphilly Study	IS	median 13 yrs	ELISA	tertile	HR	Yes
Zakai-2	2017	USA	1052 (530/522)	65.1	The REGARDS Study	HS	median 5.8 yrs	ITA	tertile/per SD	HR	Yes
**Case-control studies**											
Anzej	2007	Slovenia	90 (55/35)	38.5	—	AIS	—	ITA	—	OR	NR
Kaplan	2008	USA	1944 (1944/0)	—	The WHI observational study	AIS	—	ITA	—	OR	Yes
Shi	2014	China	214 (90/124)	67.4	—	AIS	—	ITA	quartile	OR	Yes

N (F/M): number of patients (Female/Male), The REGARDS Study: The REasons for Geographic and Racial Differences in Stroke Study, The EPICOR Study: The European Prospective Investigation into Cancer and Nutrition-Italy Cohort Study, The ARIC Study: The Atherosclerosis Risk in Communities Study, The WHI observational study: The Women's Health Initiative observational study, HS: hemorrhagic stroke, IS: ischemic stroke, AIS: acute ischemic stroke, yrs: years, ELISA: enzyme-linked immunosorbent assay, ITA: immunoturbidimetric assay, SD: standard deviation, HR: hazard ratio, OR: odds ratio, NR: not reported.

The rest three studies were case-control studies. They were published from 2007 to 2014, and were from 3 countries. A total of 2,248 patients were included (mean 749). All the three studies examined the OR of higher D-dimer level for acute ischemic stroke. The ORs were adjusted in two studies, while in the other study it is unclear whether the OR was adjusted or not.

### Pooled HR in prospective studies

**Figure 2:**
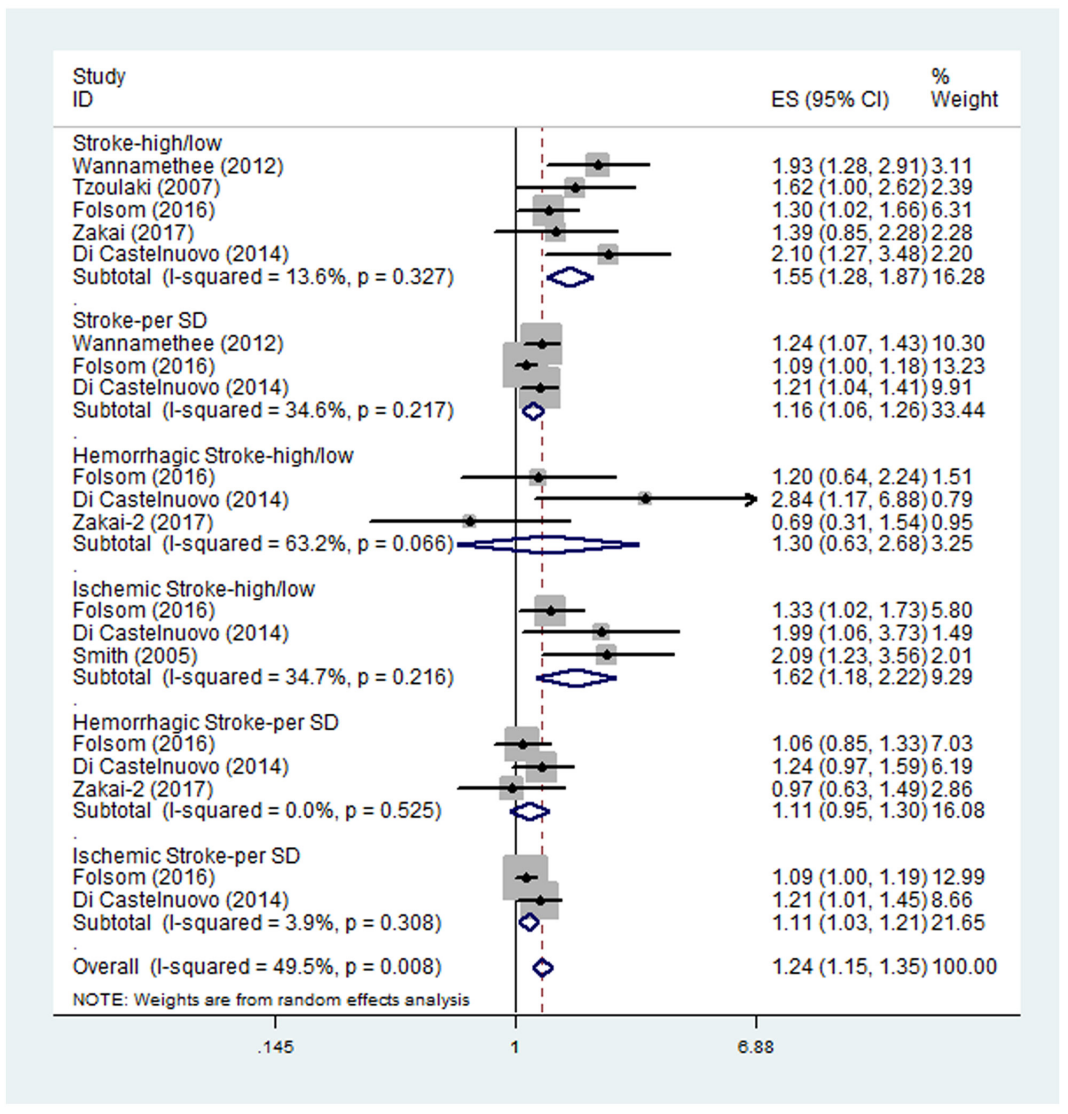
Pooled hazard ratios (HRs) of higher D-dimer level or per SD increase in log D-dimer for all types of stroke, ischemic stroke and hemorrhagic stroke

Five studies examined the HR of higher D-dimer level for all types of stroke [10, 17, 18, 30, 33]. The pooled HR of the five studies was 1.55 (95% CI, 1.28-1.87) (Figure 2), suggesting that higher D-dimer level was associated with higher risk of stroke. No significant between-study heterogeneity was found (I^2^ = 13.6%, p=0.327). Three of the five studies also examined the HR of all types of stroke per SD increase in log D-dimer [17, 18, 33]. The pooled HR of the three studies was 1.16 (95% CI, 1.06-1.26). No significant between-study heterogeneity was found (I^2^ = 34.6%, p=0.217). Subgroup analysis results were shown in Table 2. All the HRs in the subgroups were statistically significant.

Table 2Summary of meta-analysis results

**Table:** 

Prospective studies	N of studies	Pooled HR (95% CI)	p value	Heterogeneity (I^2^, P)	Conclusion	Publication bias
**Stroke-high/low**						
Total	5	1.55 (1.28-1.87)	<0.001	13.6%, 0.327	positive	0.462
Ethnicity (Europe-white)	3	1.87 (1.44-2.44)	<0.001	0.0%, 0.752	positive	—
Ethnicity (USA-with black)	2	1.32 (1.06-1.64)	0.014	0.0%, 0.812	positive	—
Sex (F/M>1)	3	1.47 (1.12-1.91)	0.005	29.0%, 0.245	positive	—
Sex (F/M<1)	2	1.79 (1.31-2.45)	<0.001	0.0%, 0.586	positive	—
Sex (male)	1	1.93 (1.28-2.90)	—	—	positive	—
Measurement (ITA)	3	1.47 (1.12-1.91)	0.005	29.0%, 0.245	positive	—
Measurement (ELISA)	2	1.79 (1.31-2.45)	<0.001	0.0%, 0.586	positive	—
Cut-off (top quintile vs bottom)	2	1.32 (1.06-1.64)	0.014	0.0%, 0.812	positive	—
Cut-off (top quartile vs bottom)	1	2.10 (1.27-3.48)	—	—	positive	—
Cut-off (top tertile vs bottom)	2	1.79 (1.31-2.45)	<0.001	0.0%, 0.586	positive	—
**Stroke-per SD**						
Total	3	1.16 (1.06-1.26)	0.001	34.6%, 0.217	positive	1
Ethnicity (Europe-white)	2	1.23 (1.10-1.36)	<0.001	0.0%, 0.819	positive	—
Ethnicity (USA-with black)	1	1.09 (1.00-1.18)	—	—	positive	—
Sex (F/M>1)	2	1.13 (1.02-1.24)	0.014	28.8%, 0.236	positive	—
Sex (male)	1	1.24 (1.08-1.44)	—	—	positive	—
Measurement (ITA)	2	1.13 (1.02-1.24)	0.014	28.8%, 0.236	positive	—
Measurement (ELISA)	1	1.24 (1.08-1.44)	—	—	positive	—
**Ischemic Stroke-high/low**						
Total	3	1.62 (1.18-2.22)	0.003	34.7%, 0.216	positive	1
Ethnicity (Europe-white)	2	2.05 (1.37-3.07)	0.001	0.0%, 0.907	positive	—
Ethnicity (USA-with black)	1	1.33 (1.02-1.73)	—	—	positive	—
Sex (F/M>1)	2	1.46 (1.05-2.05)	0.026	25.4%, 0.247	positive	—
Sex (male)	1	2.09 (1.23-3.56)	—	—	positive	—
Measurement (ITA)	2	1.46 (1.05-2.05)	0.026	25.4%, 0.247	positive	—
Measurement (ELISA)	1	2.09 (1.23-3.56)	—	—	positive	—
**Ischemic Stroke-per SD**						
Total	2	1.11 (1.03-1.21)	0.01	3.9%, 0.308	positive	1
**Hemorrhagic Stroke-high/low**						
Total	3	1.30 (0.63-2.68)	0.484	63.2%, 0.066	negative	1
Ethnicity (Europe-white)	1	2.84 (1.17-6.87)	—	—	positive	—
Ethnicity (USA-with black)	2	0.97 (0.57-1.64)	0.897	12.3%, 0.286	negative	—
Sex (F/M>1)	3	1.30 (0.63-2.68)	0.484	63.2%, 0.066	negative	—
Measurement (ITA)	3	1.30 (0.63-2.68)	0.484	63.2%, 0.066	negative	—
**Hemorrhagic Stroke-per SD**						
Total	3	1.11 (0.95-1.30)	0.177	0.0%, 0.525	negative	1
Ethnicity (Europe-white)	1	1.24 (1.00-1.65)	—	—	positive	—
Ethnicity (USA-with black)	2	1.04 (0.85-1.27)	0.698	0.0%, 0.72	negative	—
Sex (F/M>1)	3	1.11 (0.95-1.30)	0.177	0.0%, 0.525	negative	—
Measurement (ITA)	3	1.11 (0.95-1.30)	0.177	0.0%, 0.525	negative	—

**Table:** 

Case control studies	N of studies	Pooled HR (95% CI)	p value	Heterogeneity (I^2^, P)	Conclusion	Publication bias
Total	3	2.06 (1.08-3.96)	0.029	68.3%, 0.043	positive	0.296
Western countries	2	2.08 (0.72-6.04)	0.178	80.5%, 0.023	negative	—
China	1	2.32 (1.12-4.81)	—	—	positive	—
Sex (mixed)	2	2.86 (1.63-5.03)	<0.001	0.0%, 0.376	positive	—
Sex (male)	1	1.30 (0.92-1.83)	—	—	negative	—
Measurement (ITA)	3	2.06 (1.08-3.96)	0.029	68.3%, 0.043	positive	—

N number, HR hazard ratio, CI confidence interval, OR odds ratio, F/M>1 female/male>1, F/M<1 female/male<1, ITA immunoturbidimetric assay, ELISA enzyme-linked immunosorbent assay, SD standard deviation.

Three studies examined the HR of higher D-dimer level for ischemic stroke [17, 18, 35]. The pooled HR of the three studies was 1.62 (95% CI, 1.18-2.22) (Figure 2), suggesting that higher D-dimer level was associated with higher risk of ischemic stroke. No significant between-study heterogeneity was found (I^2^ = 34.7%, p=0.216). The subgroup analyses were shown in Table 2 and all the HRs in the subgroups were statistically significant. Two of the three studies also examined the HR of ischemic stroke per SD increase in log D-dimer [17, 18]. The pooled HR of the two studies was 1.11 (95% CI, 1.03-1.21). No significant between-study heterogeneity was found (I^2^ = 3.9%, p=0.308).

Three studies examined the HR of higher D-dimer level for hemorrhagic stroke [16–18]. The pooled HR of the three studies was 1.30 (95% CI, 0.63-2.68) (Figure 2), suggesting no significant association between higher D-dimer level and the risk of hemorrhagic stroke. Significant heterogeneity was found between the studies (I^2^ = 63.2%, p=0.066). Sensitivity analysis revealed that the study by Di Castelnuovo et al. [18] contributed greatly to the heterogeneity. After removing this study, the heterogeneity turned to 12.3% however the pooled HR remained not statistically significant (HR 0.97; 95% CI, 0.57-1.64). The three studies also examined the HR of hemorrhagic stroke per SD increase in log D-dimer, with the pooled HR of 1.11 (95% CI, 0.95-1.30). No significant between-study heterogeneity was found (I^2^ = 0.0%, p=0.525). The subgroup analyses were shown in Table 2. Most of the HRs in the subgroups were still not statistically significant.

### Pooled OR in case-control studies

**Figure 3:**
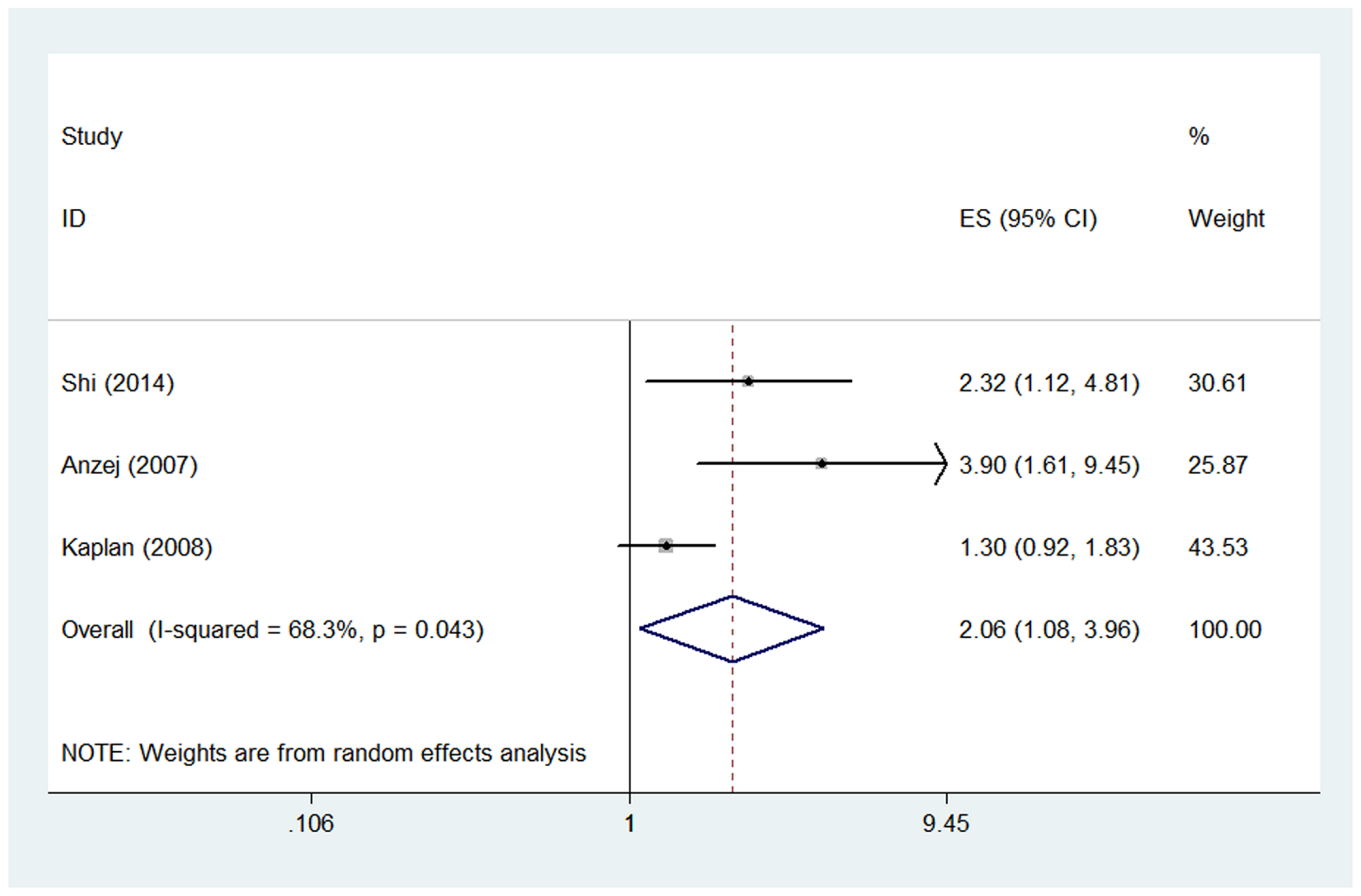
Pooled odds ratio (OR) of higher D-dimer level for acute ischemic stroke

Three studies examined the OR of higher D-dimer level for acute ischemic stroke [29, 32, 34]. The pooled OR of the three studies was 2.06 (95% CI, 1.08-3.96) (Figure 3), suggesting that higher D-dimer level was associated with higher risk of acute ischemic stroke. Significant heterogeneity was found between the studies (I^2^ = 68.3%, p=0.043). Sensitivity analysis revealed that the study by Kaplan et al. [29] contributed greatly to the heterogeneity. After removing this study, the heterogeneity turned to 0.0% and the pooled OR remained statistically significant (OR 2.86; 95% CI, 1.63-5.03). The subgroup analyses were shown in Table 2.

### Publication bias

**Figure 4:**
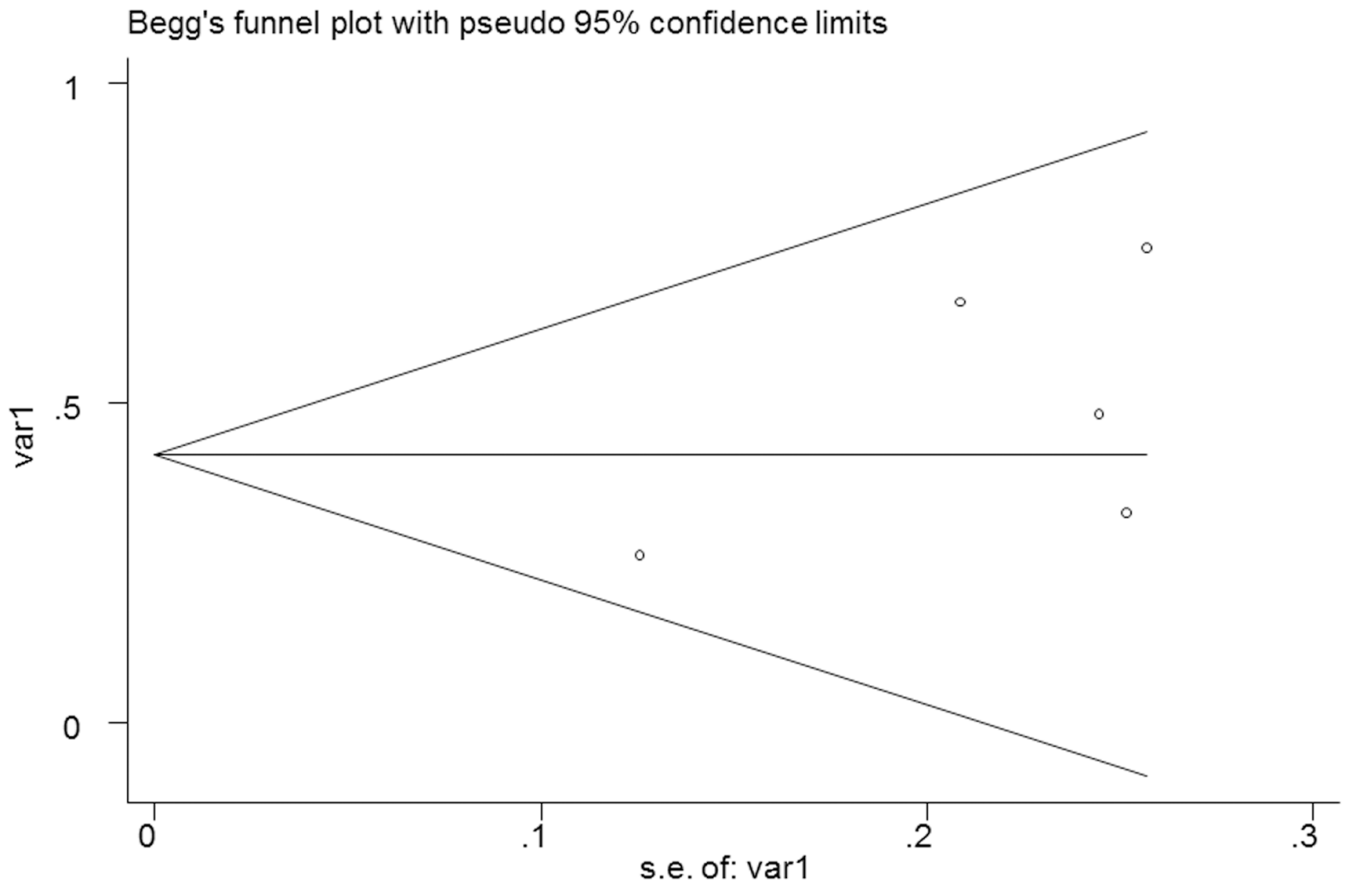
The Begg's publication bias plot of the 5 studies examining the HR of higher D-dimer level for all types of stroke (p=0.462)

No significant publication bias was found in the meta-analysis. The Begg's plot of publication bias of the 5 studies (examining the HR of higher D-dimer level for all types of stroke) was shown in Figure 4 (p=0.462).

## DISCUSSION

This study aimed to evaluate the association between D-dimer level and the risk of stroke. We performed a meta-analysis to summarize the existing evidence, and ten studies were included. To our best knowledge, this is the first meta-analysis on this issue. After pooling the prospective and case-control studies, we found that higher D-dimer level was associated with higher risk of stroke, especially ischemic stroke. As to hemorrhagic stroke, our results suggested that D-dimer was not a risk factor. In the study by Folsom et al. [17], 11,415 participants were followed up for over a median of 18 years, and the authors also concluded that D-dimer was not a risk factor for hemorrhagic stroke. But in another prospective study, the researchers suggested that elevated levels of D-dimers increase both the risk of ischaemic and hemorrhagic stroke [18]. In the study by Zakai et al, at first they found that D-dimer level was not significantly associated with hemorrhagic stroke modelled as a continuous variable or as tertiles [16]. They then added categories for the top and bottom 5% of the distribution and found a relationship with a potential threshold effect with increased risk of hemorrhagic stroke. Thus, given the different categories in the above-mentioned studies [17, 18] (quintiles and quartiles), D-dimer may be proposed as a risk factor in hemorrhagic stroke at a particular cut-off value in the future. Also, the number of studies focusing on hemorrhagic stroke is small. Therefore, more studies are needed to further explore this relationship.

Subgroup analyses were also performed according to the different characteristics of the studies. For the prospective studies, in the subgroups of all types of stroke and ischemic stroke, the HRs stayed significant, implying that D-dimer was a risk factor across different ethnicities, genders, D-dimer measurement methods and cut-off values of D-dimer. In the subgroups of hemorrhagic stroke, the HRs remained not statistically significant. For the case-control studies, the results differed across different ethnicities and genders. However, the number of studies in each subgroup was limited, and caution should be applied. More studies are warranted to verify these findings.

The underlining mechanism for the association between D-dimer level and the risk of stroke is unclear. A possible mechanism might be that D-dimer level could be a marker of systemic hypercoagulability that lead to ischemic stroke, since it may reflect the ongoing subclinical fibrin thrombus formation [36]. Folsom et al. found that higher plasma D-dimer was a risk marker for ischemic stroke, and that D-dimer is associated most strongly with cardioembolic stroke [17]. Their findings may support the above mechanism, since cardioembolic stroke is the subtype most closely linked to fibrin thrombi. D-dimer level may also reflect currently unclear thrombotic and haemostatic disorders that are related to stroke [18]. Besides, D-dimer level may reflect the heritability of the prethrombotic state [37]. Moreover, increased D-dimer may not be the causation, but a marker of a mechanism that is linked to the risk of stroke [18]. More studies are needed to elucidate the pathophysiology of such an association.

Our findings have some impact on future clinical and research work. First and foremost, D-dimer may help to screen patients at risk of stroke. Secondly, D-dimer may be used for clinical diagnosis of stroke. Besides, it may offer insights into future primary prevention strategies for stroke. Furthermore, it may be worthwhile to study the benefit of lowering levels of D-dimer in high risk populations. Also, it may help to identify patients that could benefit more from agents targeted at haemostasis rather than platelet function.

Apart from its role as a risk factor of stroke, D-dimer could also be used in other aspects among stroke patients. Recent studies have shown that D-dimer levels were different in different subtypes of acute ischemic stroke [38, 39]. Its level is the highest in cardio-embolic stroke and lowest in lacunar subtype [39]. Many studies have demonstrated that, in patients with acute ischemic stroke, higher level of D-dimer was associated with higher risk of short-term diffusion weighted-MRI defined recurrence [39], poorer functional outcome [9, 40], and higher risk of short-term mortality [41]. In patients with hemorrhagic stroke, higher level of D-dimer was also identified to predict poorer functional outcome and higher risk of short-term mortality [42, 43]. Besides, in patients with traumatic brain injury, antifibrinolytic treatment could reduce progressive hemorrhagic injury and improve outcomes [44, 45]. Thus, D-dimer may help explore new therapeutic targets for stroke in the future. Therefore, D-dimer is a promising marker which may be used in the primary prevention, diagnosis, treatment and prognosis for stroke patients. Furthermore, D-dimer is readily accessible and inexpensive for measurement.

Significant between-study heterogeneity was present among the studies examining the HR of higher D-dimer level for hemorrhagic stroke and the studies examining the OR of higher D-dimer level for acute ischemic stroke. Sensitivity analyses were performed and identified the individual studies that contributed greatly to the heterogeneities. After removing the studies, the heterogeneities turned non-significant and the pooled results remained almost the same. Potential sources of heterogeneity may be from different patient races, sex distinction, different follow-up time, different measurement methods of D-dimer and different cut-off values of D-dimer.

One of the strengths of this meta-analysis is that it included the latest large prospective studies. However, it still suffers from several limitations. Firstly, the meta-analysis was based on a small number of studies, so the results should be interpreted with caution. Secondly, the characteristics of the patients in each study were not all the same, such as different sex compositions, different follow-up time, and different cut-off values of D-dimer. Furthermore, significant between-study heterogeneity was present in some settings. Although random effect models were used to minimize the effects on results, more studies are still needed to verify these findings. Besides, although no significant publication bias was found in our meta-analysis, it was a major concern for all meta-analyses and should not be completely excluded.

In conclusion, our results suggested that higher D-dimer level was associated with higher risk of stroke, especially ischemic stroke. As a readily accessible and inexpensive marker, D-dimer is promising in the primary prevention for stroke. However, due to the limited number of studies, more studies are warranted to further verify our results.

## MATERIALS AND METHODS

### Search strategy

We followed the developed guidelines for systematic reviews and meta-analyses in performing our study [46]. PubMed, Web of Science, EMBASE and Cochrane Library were searched for potentially eligible literature (last update on Aug 2ed, 2017). The following keywords were used: ‘D-dimer’ AND (‘Stroke’ OR ‘Brain Ischemia’ OR ‘Brain Infarction’ OR ‘Cerebral Infarction’ OR ‘Intracranial Hemorrhage’ OR ‘Cerebral Hemorrhage’) AND (‘Risk’ OR ‘Risk Factors’ OR ‘Relative Risk’ OR ‘Odds Ratio’. Reference lists of relevant studies were also screened for additional literature. Languages were restricted to English and Chinese.

### Study selection

The study selection process was performed by two reviewers (JZ and YS) independently, with any disagreements being discussed. Titles and abstracts of literature were screened first, and then potentially eligible studies were evaluated in full text. Studies were considered for inclusion if they met all of the following criteria: (1) prospective observational studies or case-control studies; (2) blood samples were collected at baseline or after admission to assess the level of D-dimer; (3) for prospective observational studies, patients were followed up for stroke events (either ischemic or hemorrhagic); for case-control studies, stroke patients were compared with controls; (4) enough data was reported to estimate the association between D-dimer level and the risk of stroke. Letters, case reports, reviews, conference abstracts, unrelated articles, and studies without enough data were excluded. If multiple studies were performed in the same center and the patients overlapped, the study with the largest sample size and longest follow-up time was included.

### Data extraction

Relevant data were extracted from the eligible studies by two researchers (JZ and YS) independently, and disagreements were resolved by consensus. The primary data was hazard ratio (HR) or odds ratio (OR) with 95% confidence interval (CI), or the data that could be used to calculate the HR or OR with 95% CI. Estimates calculated form multivariate analyses were extracted over those calculated from univariate analyses. The characteristics of the studies and patients were also extracted, including first author, publication year, country, study design, follow-up time, patients’ number, sex of patients, mean or median age of patients and so on.

### Statistical analysis

The logHR (or logOR) and variance were calculated from the HR (or OR) and 95% CI, and were used for aggregation of data. Forest plots were constructed to estimate the association between D-dimer level and the risk of stroke. The pooled HR (or OR) was regarded significant if the p value was less than 0.05 and the 95% CI did not overlap 1. Subgroup analyses were also performed according to patient ethnicity, gender, D-dimer measurement method and cut-off value of D-dimer. The heterogeneity between studies was also assessed, with I^2^>50% or p<0.10 indicating significant heterogeneity [47]. Random effect models were used in pooling the studies no matter whether heterogeneity exited, since some heterogeneity among studies was expected due to differences in patient characteristics, D-dimer level measuring and other study differences [48]. If heterogeneity was present, sensitivity analysis was performed to evaluate the contribution of each study to heterogeneity by excluding individual studies one at a time. Publication bias was assessed by Begg's test, with p>0.05 indicating no significant publication bias. All the above mentioned statistical analyses were performed by STATA 11.0 (STATA Corporation, College Station, TX).
